# A cautionary note on ignoring polygenic background when mapping quantitative trait loci via recombinant congenic strains

**DOI:** 10.3389/fgene.2014.00068

**Published:** 2014-04-02

**Authors:** J Concepción Loredo-Osti

**Affiliations:** Department of Mathematics and Statistics, Memorial UniversitySt. John's, NL, Canada

**Keywords:** misspecified genetic models, bootstrapping mixed models, recombinant congenic strains, ignoring random effects, mapping quantitative trait loci

## Abstract

In gene mapping, it is common to test for association between the phenotype and the genotype at a large number of loci, i.e., the same response variable is used repeatedly to test a large number of non-independent and non-nested hypotheses. In many of these genetic problems, the underlying model is a mixed model consistent of one or very few major genes concurrently with a genetic background effect, usually thought as of polygenic nature and, consequently, modeled through a random effects term with a well-defined covariance structure dependent upon the kinship between individuals. Either because the interest lies only on the major genes or to simplify the analysis, it is habitual to drop the random effects term and use a simple linear regression model, sometimes complemented with testing via resampling as an attempt to minimize the consequences of this practice. Here, it is shown that dropping the random effects term has not only extreme negative effects on the control of the type I error rate, but it is also unlikely to be fixed by resampling because, whenever the mixed model is correct, this practice does not allow to meet some basic requirements of resampling in a gene mapping context. Furthermore, simulations show that the type I error rates when the random term is ignored can be unacceptably high. As an alternative, this paper introduces a new bootstrap procedure to handle the specific case of mapping by using recombinant congenic strains under a linear mixed model. A simulation study showed that the type I error rates of the proposed procedure are very close to the nominal ones, although they tend to be slightly inflated for larger values of the random effects variance. Overall, this paper illustrates the extent of the adverse consequences of ignoring random effects term due to polygenic factors while testing for genetic linkage and warns us of potential modeling issues whenever simple linear regression for a major gene yields multiple significant linkage peaks.

## 1. Introduction

For more than four decades, linear mixed models have been used in a wide range of applications because of their conceptual simplicity and flexibility to accommodate correlated sources of variation as well as fixed regressors. A generic linear mixed model can be written as

(1) y=Xβ+Zγ+e

where **X** and **Z** are known incidence matrices, β is a vector of unknown fixed regression coefficients, γ is a vector of random effects, and **e** is the vector of errors. It is also common to assume that γ and **e** are independent and both have null expectation and finite variances. In many situations, either intentionally or unintentionally, the statistical analysis is carried out ignoring the term **Z**γ in the model. This practice, although recognized as inefficient, has been thought to be harmless whenever the interest resides solely on a subset of the regression coefficients with the remaining parameters of the model deemed as nuisance. This thought seems to be mostly based on the fact that β^*o*^ = (**X**′**X**)^−1^**X**′**y** is still an unbiased and consistent estimator of β. However, it is well known that ignoring **Z**γ and using ordinary least squares, results in an estimator of Var(β^*o*^) that is biased and inconsistent as well as non-independent of β^*o*^ (Dhymes, 1978). Of course, this will affect the distribution properties associated with β^*o*^ under normality or, otherwise, the asymptotic properties of its distribution. It has been suggested that this problem can be mitigated if testing is done through resampling. However, the adverse consequences of dropping the random term from the mixed model is unlikely to be fixed by the use of resampling methods. In this paper, a specific application to genetic mapping via recombinant congenic strains (RCS) of experimental animals is used to illustrate this. Briefly speaking, genetic mapping can be seen as a problem in which the association of one dependent variable (the phenotype) with a large number of potential explicative variables (the marker genotypes) is tested one-by-one or by taking a very small number of markers at once. An RCS panel is a replicable mapping population for which animals within the same strain are considered to be genetically identical and related to different degrees with animals from other strains. Such an inter-strain relationship results in what is known as the genetic background effect and, whenever this effect is understood as the result of the addition of many components of minuscule effect, the inclusion of a random effects term in the model would be the natural way to account for it.

A mouse panel of RCS is obtained by mating mice from two genetically distinct inbred strains (a donor strain and a recipient strain) followed by two or more rounds of backcrossing to the recipient strain and subsequent sister × brother mating without selection for particular markers or phenotypes for a minimum of 20 generations. The genetic resolution of the panel is controlled by the number of backcrossing rounds. Because of this construction, each strain of an RCS panel can be thought of as an inbred strain in which segments of random length from the genome of a recipient strain have been replaced with the corresponding segments from a donor strain. The main consequence of this breeding scheme is that non-linked genes controlling the same trait are separated and fixed in haplotypes of different strains, allowing the possibility of studying them individually. The standard RCS panel uses two backcross generations and, consequently, the total length of the segments from recipient strain constitute on average the 87.5% of the genome of each strain; the remaining 12.5% represents the total expected length of the replaced genome segments. Without loss of generality, this is the type of RCS considered in this paper. For a more comprehensive description of the RCS and their use in gene mapping see Démant and Hart (1986), Moen et al. (1992), and Fortin et al. (2001b, 2007). Once the RCS panel have been established, the whole panel is genotyped to obtain full characterization of the genome of each strain. Each genotype data set can then be used for the analysis of all individuals of the same strain; this is an important money-saving feature of the design since it does not require of re-genotyping each individual because, except for *de novo* mutations, all pups from the same strain are genetically identical.

Although most mouse geneticists agree that RCS are a powerful resource to map loci associated with complex traits, there is some disagreement on how to do the analysis. Originally, when the use of RCS for genetic mapping was proposed, the core idea was to look into the stain distribution pattern with respect to a phenotype of interest and identify the strain that exhibited the largest deviation from the other strains in the RCS panel and subsequently cross it with the recipient strain to obtain *F*_1_ and *F*_2_ progenies to be analyzed by standard methods (Démant and Hart, 1986; Fortin et al., 2001b). Two examples of the application of this approach are reported in Fortin et al. (2001a) and Müllerová and Hozák (2004). The problem is that contrasting phenotypes from *F*_1_ mice versus the ones from the recipient strain will only be effective for dominant traits, while the power for additive traits will be diminished and lost completely for recessive traits. On the other hand, the analysis of the *F*_2_ mice requires new genotyping, which not only defeats the economic advantages of having developed RCS, but more importantly, because every *F*_2_ individual has different genotype, this approach is not suited for complex quantitative traits when a single measurement may not be reliable enough to determine the phenotype (Moen et al., 1992). Alternatively, there is a designs consisting of taking a sample of mice from each strain and analyzing the whole panel together. Although this approach does not require additional genotyping and has the potential for making more efficient use of the phenotypic variation, also opens more room for analysis pitfalls if the proper model is not used. For example, Joober et al. (2002) uses a QTL mapping procedure equivalent to simple linear regression at the markers ignoring genetic background which, as pointed by Palmer and Airey (2003), it may result in false positive rates far in excess of the nominal value, even when Bonferroni corrections are used. Another common way to address the problem is to use strain averages as the phenotype and treat the panel of means as a backcross dataset for analysis purposes. This is essentially the “interval mapping” procedure proposed by Shao et al. (2010) and equivalent to the one used by Thifault et al. (2008). This approach may substantially reduce the power for RCS panels with reduced number of strains and it does not deal with the fact that the strains, related because their background, may not have the same kinship degree at genomic level and consequently the phenotype means may be not only non-independent but heteroscedastic, as well. Lee et al. (2006) and Camateros et al. (2010) extend the simple linear regression to account for the genetic background by adding a fixed factor (“background proportion” in the first paper; “background indicator” in the second). Although better than ignoring the background, from the genetics standpoint, it is difficult to justify the plausibility of a fixed effects model under the assumption that the background effect is the result of the additive action of many genes of minuscule effect. In fact, I argue that the natural way to model such a background effect consistent with the principles outlined by Fisher (1919) is through the inclusion of a random effects term in the model as implemented in Di Pietrantonio et al. (2010). In this paper, I describe in detail a procedure for the analysis of a quantitative trait locus (QTL) that models the genetic background (assumed to be of polygenic nature) as a random effect term and use this to show how the omission of such a term in the model leads to conclusions that are wrong and inconsistent with the data.

## 2. Models

### 2.1. The naive QTL model for an RCS panel

In its simplest form, at each marker position *m*, *m* = 1, 2, …, *M*, the RCS/QTL model for the *i*th individual, *i* = 1, 2, …, *n*, can be written as

(2) $$y_{i}=\mu +q_{im}\xi_{m}\;+e_{i}$$

where *y*_*i*_ denotes the phenotype for the *i*th individual, ξ_*m*_ denotes the major locus effect associated with the *m*th marker, *q*_*im*_ is the indicator of the BB genotype at the *m*th position which is determined by the RCS data, and the *e*_*i*_s are a set of independent random variables with distribution 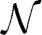
(0, σ^2^) (AA and BB are the genotypes of the donor and recipient parental strain, respectively). Of course, under an oligogenic model, at most, a handful of ξ_*m*_s should be different from zero. In fact, it is common practice that at the first screening, the estimation is carried out by regression at each marker under the assumption of only one major gene. When the presumption of a dense enough genotyping marker panel is not correct, procedures like modified interval mapping can be used instead. Variations of the problem include conditioning on a given set of markers. The salient feature of this design is that, at the *m*th marker position, one looks across the RCS panel and classifies each strain as either AA or BB, since under the model (Equation 2), this is the only source of genetic variation when estimating ξ_*m*_. However, this model ignores the fact that individuals from the same strain are genetically identical (assuming no new mutation at the locus under scrutiny), and strains with the same ancestral background share large portions of their genome so that even without the involvement of a major gene, there is more likely to be reduced variation within strains. In a nutshell, regression mapping works by testing the association of the phenotype with the observed genotype at each marker location so that finding significant linkage at any position implies testing the *M* null hypotheses, ξ_*m*_ = 0. Clearly, most of these hypotheses as well as their test statistics are not independent. This may lead to problems in the control of the type I error rate if multiple testing is not addressed properly. Another irregularity results from the fact that with a dense genotyping panel the number of tested hypotheses can by far exceed the sample size. Because of these considerations, *p*-value estimation by resampling of residuals has been seen as a plausible alternative. For this paper, the problem is addressed through bootstrap.

#### 2.1.1. Computation of *p*-values

The estimation of genome-wide corrected *p*-values by resampling requires that under the null hypothesis: (i) each resample is taken from an exchangeable distribution, (ii) the variation of the original sample is preserved through all resamples, and (iii) the genome-wide baseline for the test statistics at each position is the same. The first two requirements are standard for resampling in regression (Davison and Hinkley, 1997; Anderson and Ter Braak, 2003). The last requirement is imposed to ensure that the uncorrected *p*-values across the genome are comparable (this is particularly important when there are missing genotype data). One way to estimate corrected *p*-values is to select an ensemble of test statistics whose marginal distribution is the same when the model does not contain any major locus.

Since under model (Equation 2) and the hypothesis of no major gene, the distribution of **y** = (*y*_1_, *y*_2_, …, *y*_*n*_)′ is exchangeable, resampling from the raw observations will also preserve the variation through the pseudo-observations. This means that in the absence of non-genetic regressors or other non-oligogenic factors, resampling the raw phenotypes either by permutation or through bootstrap will produce similar results. Furthermore, under these premises, basic sampling and hypothesis testing principles indicate that a permutation based procedure will be more efficient and powerful. However, this is not necessarily the case when the premises are removed. Should the model also contain fixed non-genetic regressors, resampling from the leverage-adjusted residuals under the null hypothesis would be a procedure that approximates exchangeability while preserving the original variation of the data. However, under this situation, resampling from leverage-adjusted residuals results in a procedure with acceptable properties only in the bootstrap case (Davison and Hinkley, 1997), while this is not longer guaranteed when resampling via permutation. The main issue is that sampling without replacement magnifies the effects of modest departures from exchangeability. Then, permuting leverage-adjusted residuals may not be good enough (even worst, it may not be valid) and we would require of a much more elaborate and computer intensive procedure to obtain residuals guaranteed to be at least weakly exchangeable so that permutation works properly [see, for example, Kherad-Pajouh and Renaud, 2010. To complete the requirements listed above regarding the possibility of missing genotypes, we propose to use the test statistic defined by the expression

(3) $$z_{m}=t_{m}\left(1-\frac{1}{4\nu_{m}}\right){\left(1+\frac{t_{m}^{2}}{2\nu_{m}}\right)}^{-\frac{1}{2}}\text{ where }t_{m}=\frac{\left|{\hat{\xi }}_{m}\right|}{{\hat{\sigma }}_{{\hat{\xi }}_{m}}}$$

and ${\hat{\xi }}_{m}$ is the ordinary least squares estimate of ξ_*m*_, *m* = 1, 2, …, *M*, i.e., *z*_*m*_ is just *t*_*m*_, our familiar *t*-statistic with ν_*m*_ degrees of freedom, transformed into a *z*-score (ν_*m*_ may vary slightly from marker to marker due to missing data). Another option would be a modified *t*-statistic *t*′_*m*_ in which the *m*th estimate of variance *s*^2^_*m*_ used to compute ${\hat{\sigma }}_{{\hat{\xi }}_{m}}^{2}$ is replaced by *s*^2^_0_, the estimate under the null hypothesis. With no missing genotypes the use of any of *z*_*m*_, *t*′_*m*_, and *t*_*m*_ would yield approximately the same *p*-value estimates.

#### 2.1.2. Bootstrap procedure for simple linear regression at the markers

The following bootstrap procedure computes the genome-wide corrected *p*-values for model (Equation 2) with the test statistic (Equation 3):

Step 1. At each marker position, *m*, fit the simple linear regression at the markers model (Equation 2), use (Equation 3) to compute the test statistic *z*_*m*_, and obtain the genome-wide set of statistics 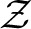
_*M*_ = {*z*_*m*_, *m* = 1, 2, …, *M*}. Also, set the genome-wide acceptance count vector to zero.
Step 2. Sample with replacement from the raw vector of phenotypes, **y** ∈ ℝ^*n*^, to obtain y^*^ ∈ ℝ^*n*^, a bootstrapped full replica of **y**, and use this vector to compute z^*^_max_ = max {z^*^_m_}, where z^*^_m_, *m* = 1, 2, …, *M*, is the test statistic at the *m*th locus, computed by using y^*^, the vector of the pseudo-observations, instead of the original vector of phenotypes.
Step 3. For each *z*_*m*_ in 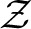
_*M*_, if *z*_*m*_ ≤ z^*^_max_, add a unit to the *m*th entry of the acceptance count vector.
Step 4. Repeat steps 1 and 2 *R* times and then compute the estimate of the vector of *p*-values by dividing the acceptance count vector by *R*.

This resampling scheme can be seen as an adaptation of a regular regression residuals bootstrapping procedure (Davison and Hinkley, 1997), coupled with Roy's union-intersection principle (Roy, 1953) to control for the genome-wide type I error rate. When applied to the analysis of the RCS panel, this procedure is valid when there is only one observation per strain or when the within-strain variation is negligible. Otherwise, a random term in the model has been neglected and, regardless of ${\hat{\xi }}_{m}$ being an unbiased estimator of ξ_*m*_, the exchangeability requirement cannot be met and the most likely consequence would be an inflated type I error rate. In fact, as per arguments given by Churchill and Doerge (1994) and Churchill and Doerge (2008), this statement is correct not only for the bootstrap and RCS, but also for permutation test procedures applied to any study design involving replicable mapping populations because, as for bootstrap, the Fisher (1935) principle of permutation also relies on exchangeability. For simple experimental designs such as an intercross or a backcross mating, the individual units can safely be assumed to be exchangeable. However, it would be wrong to assume exchangeability for more complicated designs, like advanced intercross, heterogeneous stocks and RCS.

### 2.2. The QTL mixed model for an RCS panel

The previous simple linear model (Equation 2) generalizes to a model of the form:

(4) $$y=X\beta +Z\gamma +q_{m}\xi_{m}+e$$

where **y** represents the phenotype vector, **q**_*m*_ is a vector with each entry being an indicator variable of the genotype BB at the marker position *m* with ξ_*m*_ being its associated effect (major gene effect), γ is a random effects vector associated with the genetic background with E(γ) = **0** and Var(γ) = σ^2^_γ_ Δ_1_, with σ^2^_γ_ > 0 and Δ_1_, a positive-definite matrix, both assumed to be constant, although unknown, **X** is a matrix of fixed covariates and its corresponding parameter vector β, **e** is a vector of independent and identically distributed random variables representing the error term with E(**e**) = **0** and Var(**e**) = σ^2^ **I**. Up to a multiplicative constant, Δ_1_ is a function of the length of the segments identical by descent shared amongst strains. For an established RCS panel there are only two possible identity states between pairs of strains at a given locus: either (i) all four alleles are identical by descent (Δ_1_ is the matrix holding the pairwise probabilities for this state), or (ii) the strains have different allelic forms and thus identical by descent only amongst themselves. So an estimator of Δ_1_ with “a high degree of precision” can be reached. Such an estimator uses only genomic information and does not involve **y**, so when estimating the parameters, one can assume that Δ_1_ is given. Another option is to take the entries of Δ_1_ as the expected value of the proportion of the genome shared identical by descent between the respective strains under the RCS panel construction described above, i.e.,

(5) $$\delta_{1}{}_{ij}=\{\begin{array}{ll} 1 & \text{if }i=j\\ \frac{15}{16} & \text{if }i\text{ and }j\text{ have the same background}\\ \frac{1}{16} & \text{if }i\text{ and }j\text{ have different backgrounds}. \end{array}$$

This option, although not the most efficient, does capture the main features of the design and yields a variance structure for the random effects vector that can be exploited in the implementation of the resampling algorithm. For example, if all the strains in the panel under scrutiny have the same background and the simplified expectation-based Δ_1_ is used, then the distribution of the vector of random effects is exchangeable. Nonetheless, replacing a genomic-based Δ_1_ estimate by its theoretical expectation (Equation 5) implies ignoring important information regarding the correlation of the additive polygenic effects associated to the genetic background.

#### 2.2.1. Estimation

The estimation for the mixed linear model has been extensively discussed in the literature (Harville, 1977; Henderson, 1986). Here we develop an application of these standard methods to the RCS design. Without loss of generality, let us consider the linear mixed model (Equation 1) with Var(γ) = σ^2^_γ_ Δ_1_ and Var(**e**) = σ^2^**I**. Thus

$$\text{E}\left(y\right)=X\beta \text{ and }\mathrm{var}\left(y\right)=\sigma^{2}\left(ZGZ^{\prime }+I\right)=\sigma^{2}\Sigma$$

where **G** = λ Δ_1_ and $\lambda =\frac{\sigma_{\gamma }^{2}}{\sigma^{2}}$, i.e., λ represents the signal-to-noise ratio. Under the assumption of no major gene and only polygenic background, λ is related to the heritability coefficient. When **G** is known, the best linear unbiased estimator of β and the best linear unbiased predictor of γ (also known as a shrinkage estimator) can be written as

$$\tilde{\beta }={\left(W^{\prime }W\right)}^{-}W^{\prime }v\text{ and }\hat{\gamma }=GZ^{\prime }\Sigma^{-\frac{1}{2}}(v-W\tilde{\beta }),$$

respectively, where **W** = $\Sigma^{-\frac{1}{2}}$**X**) and **v** = $\Sigma^{-\frac{1}{2}}$**y**). Also

$$\begin{array}{l} {\hat{\sigma }}^{2}=\frac{1}{N-\text{rank}(W)}{\left(v-W\tilde{\beta }\right)}^{\prime }(v-W\tilde{\beta })\\ {\hat{\sigma }}_{\gamma }^{2}=\frac{1}{\text{rank}(G)}({\hat{\gamma }}^{\prime }G^{-1}\hat{\gamma }+{\hat{\sigma }}^{2}\text{tr}(G^{-1}C)) \end{array}$$

with

$$C={\left(Z^{\prime }MZ+G^{-1}\right)}^{-1}\text{ and }M=I-X{\left(X^{\prime }X\right)}^{-1}X^{\prime }.$$

Notice that the previous expressions cannot be computed unless the signal-to-noise ratio, λ, is known. A situation of a more practical interest is an iterative procedure on which λ is replaced by its estimate and, once that the estimates of σ^2^ and σ^2^_γ_ have been updated, a refinement of the estimate of λ is obtained and so on. This iterative procedure will result in a $\tilde{\beta }$ and $\hat{\gamma }$ that are no longer linear, nonetheless, they preserve most of the desirable properties present in their linear counterpart (Jiang, 1998).

#### 2.2.2. Mixed model resampling scheme

Let us now focus our attention toward a resampling scheme appropriate for RCS data under a mixed model. By now, it is obvious that the bootstrap procedure described in the previous section will not work for the mixed model (Equation 4). A crude extension to this procedure would consist of computing

$$\hat{e}=y-X\tilde{\beta }-Z\hat{\gamma }$$

and resampling from $\hat{\gamma }$ and $\hat{e}$ to obtain γ^*^ and e^*^ so that the pseudo-observation y^*^ could be recovered as

$$y^{*}=X\tilde{\beta }+Z\gamma^{*}+e^{*}.$$

However, it is straightforward to see that these residuals are not exchangeable and they are biased toward zero. Thus, they may not adequately represent the hypothesis tested nor reflect the true variation of the model.

Alternatively, note that when β and λ are known, it follows from the model under the null hypothesis that E(**v**) = **W**β and Var(**v**) = σ^2^ **I** which implies that the distribution of the vector of residuals, ϵ = **v** − **W**β, is exchangeable. This suggests the following residuals resampling scheme:

1. given $\tilde{\lambda }$ and $\tilde{\beta }$ obtained under the mixed model without a major gene, i.e., under the null hypothesis, compute $\tilde{\Sigma }$, $\tilde{W}$ by replacing λ with $\tilde{\lambda }$ and Δ_1_ with its genomic-based estimate; then, obtain the leverage-adjusted residuals
  $$\tilde{\epsilon }=D({\tilde{\Sigma }}^{-\frac{1}{2}}y-\tilde{W}\tilde{\beta })$$
  where **D** is a diagonal matrix with each of the non-zero elements given by (1 − *h*_*ii*_)^−1^ and *h*_*ii*_ is the *i*th leverage coefficient;
2. with replacement, resample from $\tilde{\epsilon }$ ∈ ℝ^*n*^ to obtain ϵ^*^ ∈ ℝ^*n*^, its bootstrapped replica, and construct the vector of pseudo-observations as
  $$v^{*}=\tilde{W}\tilde{\beta }+\epsilon^{*}.$$

If instead of a bootstrap procedure based on leverage-adjusted residuals we want to use a residuals-based permutation procedure, then we need to extend the method of Kherad-Pajouh and Renaud (2010) to get weak exchangeability of residuals. However, when λ is estimated from the data, such an extension is not possible and we would have to rely on approximations. More research is needed to explore this direction.

Outside of a genetics context, there is a number of permutation and bootstrap procedures for mixed models whose objective is testing the components of variance (for example, Fitzmaurice et al., 2007; Sinha, 2009; Lee and Braun, 2012; Samuh et al., 2012). However, they cannot be applied in our case because we are interested in the regression coefficients (or a subset of them) and the variance of the random effects is just nuisance parameter. Incidentally, when testing the components of variance, bootstrap has the edge over most permutation procedures (Samuh et al., 2012).

#### 2.2.3. Bootstrap procedure for the mixed linear model

According to the foregoing argument, generalization to the previous bootstrap procedure to compute the genome-wide corrected *p*-values for the mixed model (Equation 4) goes as follows:

Step 0. Compute Δ_1_ from the genotype data of the RCS panel, and under the null hypothesis, obtain $\tilde{\lambda }$, $\tilde{\beta }$, $\tilde{\Sigma }$, $\tilde{W}$ and $\tilde{\epsilon }$ as described in (i) above.
Step 1. At each marker position, *m*, fit the model
  (6) $$\tilde{v}=\left(\tilde{W}\;{\tilde{\Sigma }}^{-\frac{1}{2}}q_{m}\right)\left(\begin{array}{c} \beta \\ \xi_{m} \end{array}\right)+\epsilon .$$
  Of course, this model is equivalent to model (Equation 4), the RCS/QTL mixed model, with λ replaced by $\tilde{\lambda }$. Compute the model parameter estimates with the outlined mixed model procedure as well as the test statistic set 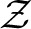 = {z_*m*_, *m* = 1, 2, …, *M*} by using Equations (6) and (3); set the acceptance count vector to zero.
Step 2. Draw a pseudo-observation v^*^ by using the proposed resampling scheme in (ii) above and fit the major gene model in model (Equation 6) with $\tilde{v}$ replaced by v^*^ to obtain the set of bootstrapped test statistics {z^*^_m_} and its associated critical value z^*^_max_ = max {z^*^_m_}.
Step 3. For each *z*_*m*_ in 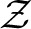, if *z*_*m*_ ≤ z^*^_max_, add a unit to the *m*th entry of the acceptance count vector.
Step 4. Repeat steps 2 and 3 *R* times and compute the *p*-value estimates by dividing the acceptance count vector by *R*.

To my knowledge, this bootstrap procedure for the analyzing a panel of RCS has not been proposed before Di Pietrantonio et al. (2010) and this paper contains the first detailed derivation and study of its properties. In fact, the resampling methods (mostly conditional permutation) applied to analyze RCS have not used mixed models, but consider the strain effect as fixed which is inconsistent with the hypothesis of a genetic background of polygenic nature or discard information by using only the estimated strain means (for example, Gill and Boyle, 2005; Thifault et al., 2008; Camateros et al., 2010).

## 3. Results

One straightforward way to show the effect of ignoring the random effects term in a mixed model is by simulation. The idea is to generate a dataset from a model that includes a random term for genetic background and noise, but is free of any major locus. Then compare the *p*-value profiles (actually, −log_10_ *p* profiles) obtained by the use of the naive model (Equation 2) as well as the mixed model (Equation 4). For this simulation study, the genotypes of an RCS panel of 36 strains that were described in Fortin et al. (2001b) were used. The panel originally had 37 lines and 625 microsatellite markers; since then, one line has died out and six markers were removed for reliability reasons. Although a much larger set of single nucleotide polymorphism markers for this RCS panel is also available, I think that this set of 619 markers is enough to show the harmful effects of fitting the wrong model on the inference. Of course, more markers will only exacerbate the problem. For this simulation experiment, six different values for the signal-to-noise ratio parameter λ were chosen (0, $\frac{1}{8}$, $\frac{1}{4}$, $\frac{1}{2}$, 1, and 2). Under a standard additive polygenic model, i.e., a model without major genes, the signal-to-noise parameter is a function of the heritability coefficient (the chosen values correspond to the heritability proportions of 0, $\frac{1}{9}$, $\frac{1}{5}$, $\frac{1}{3}$, $\frac{1}{2}$, and $\frac{2}{3}$, respectively). In every simulation run, a sample of seven individuals from each strain was simulated under the assumption of no major gene, i.e., under model (Equation 4) with ξ_*m*_ = 0 for all markers, *m* = 1, 2, …, *M*. The value of σ^2^ was fixed for all simulations to 1.175, while **X**β was fixed as a vector with 7 in all its entries. Simulations for each value of λ were run 1000 times and both methodologies, the mixed model as well as the bootstrapped naive regression at the markers were applied to the simulated datasets with 10,000 as the number of resamples for every dataset. In gene mapping studies, a significant peak is defined as the most extreme point of a region beyond the *p*-value threshold according to some pre-specified genome-wide type I error rate (Churchill and Doerge, 1994). For this study, we use a value of 0.01 or equivalently, a threshold value of 2 on a −log_10_ *p*-scale. Tables 1–3 summarize the results of these simulations. As expected, whenever there is not a polygenic term in the model (i.e., λ = 0), both methodologies produce identical results. However, the picture changes when λ > 0. In this case, it is quite obvious that ignoring the random effects term has pernicious consequences even for modest levels of λ, the signal-to-noise ratio, while the proposed mixed model method keeps the genome-wide type I error rate relatively close to the nominal value. However, the empirical type I error rates obtained by the proposed procedure seem to increase slightly with λ (Table 3). This phenomenon may be due to the fact that the makers used for mapping purposes are also used to estimate the probability of identity by descent between strains and, to a lesser extent, the fact that the the bootstrap procedure is based on residuals computed with λ and β estimated from the same data. Nonetheless, the moral of this exercise is that whenever simple regression of a major gene model produces many significant peaks, a warning flag about the model validity should be raised.

**Table 1:**
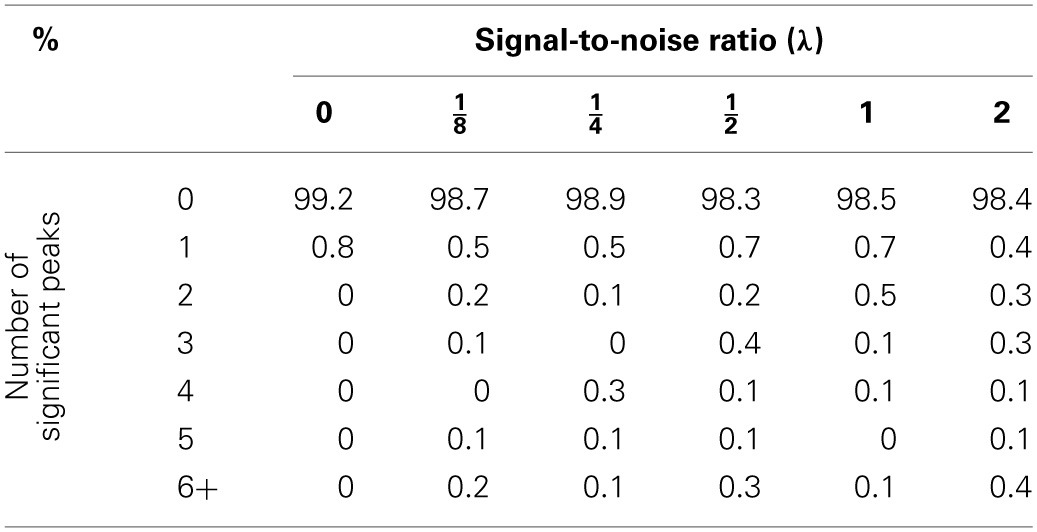
**Percentage of declared significant peaks with a bootstrap genome-wide adjusted significance level of 0.01 when the proposed mixed model methodology is used**.

Estimates based on 1000 simulated datasets for each λ.

**Table 2:**
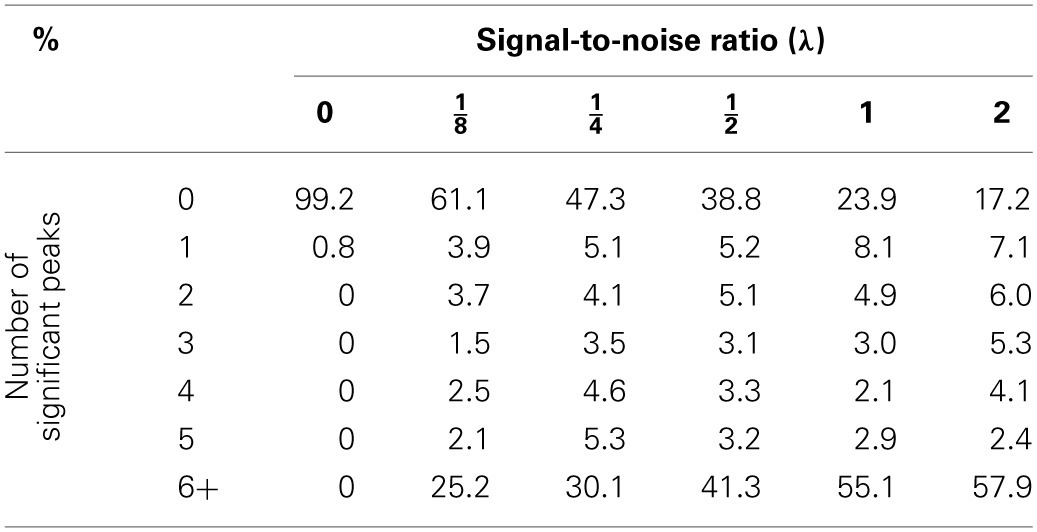
**Percentage of declared significant peaks with a bootstrap genome-wide adjusted significance level of 0.01 when a naive regression at the markers is used**.

Estimates based on 1000 simulated datasets for each λ.

**Table 3:** **Empirical genome-wide type I error rates obtained via bootstrap in the simulation study (0.01 is the nominal value and the number of simulated datasets for each λ is 1000)**.

	**Signal-to-noise ratio (λ)**					
	**0**	**$\frac{1}{8}$**	**$\frac{1}{4}$**	**$\frac{1}{2}$**	**1**	**2**
Naive regression	0.008	0.389	0.527	0.612	0.761	0.808
Mixed model	0.008	0.013	0.011	0.017	0.015	0.016

**Figure 1:**
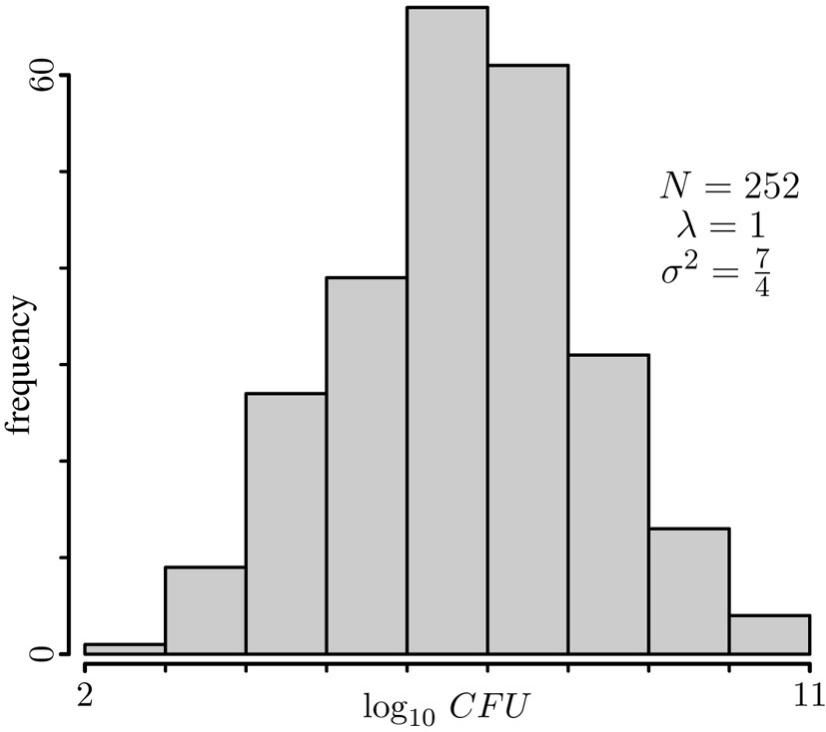
**Typical histogram of simulated data.** The *p*-value profiles of the data on this histogram were computed and plotted in Figure 2.

**Figure 2:**
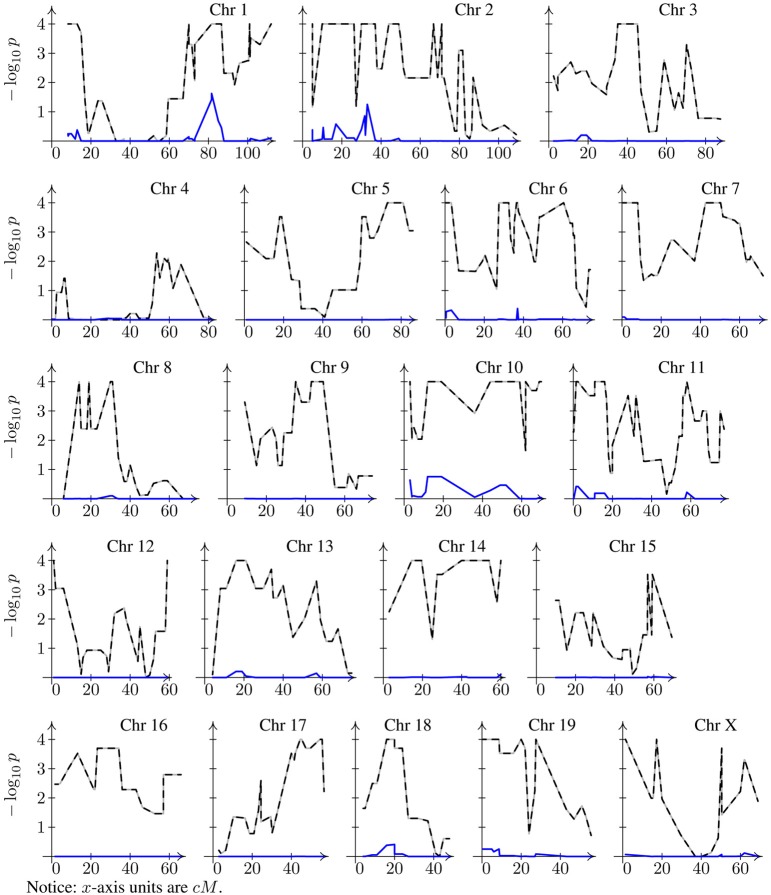
**Bootstrap genome-wide corrected *p*-value profiles.** Dashed line for naive model (Equation 2) and solid line (sometimes hardly distinguishable from the *x*-axis line) for the mixed model (Equation 6). Note that both profiles have been corrected for multiple testing.

The histogram of a typical dataset obtained by simulation from a model with polygenic effects only would look like the one shown in Figure 1. Nonetheless, for this histogram I chose a dataset for which simple linear regression produces a very large number of significant peaks. If a major locus were at play, one would expect to have a well-defined bimodal distribution, so this histogram seems consistent with the generating model of no major gene. However, when we look into the *p*-value profiles obtained through the model that ignores the genetic background term, instead of profiles consistent with the model we will have something extreme as shown by dashed lines in Figure 2. According to the profiles on this figure, one might conclude that all chromosomes have at least one significant peak, fact that does not appear to be supported by the histogram of the data, and more conclusively, this is in conflict with the generating model. If anything, it can be argued that the data distribution may seem a bit skewed, but one may expect that estimation of *p*-values via bootstrapping of residuals should not be too sensitive to this. Of course, as for bi-modality, skewness may also be caused by a mixture of distributions. However, a very strong peak, as any of the ones spotted on every chromosome, is difficult to conceive without a conspicuous bimodal distribution. Even with the use of robust regression estimates instead of the obtained by regular least squares to minimize the potential impact of outliers on the estimation, these profiles change very little (data not shown). When the missing random effects term is introduced into the model (solid blue lines in Figure 2), *p*-value profiles become consistent with the generating model. Repetition of this exercise on any other simulated datasets yields similar results, although the specific resulting profiles most likely are not be the same.

## 4. Discussion

This paper proposes a bootstrapping procedure to estimate the *p*-values under a mixed model applied to gene mapping when RCS are used. The method can be easily adapted for other replicable mapping population/designs. This procedure is a generalization of the linear regression bootstrap of residuals coupled with the union-intersection principle aimed to control the genome-wide type I error rate. A simulation study with different values of the signal-to-noise ratio unequivocally shows that when a panel of RCS is used for mapping, ignoring one random effects term in a mixed linear model can have pernicious consequences, resulting in inflated type I error rates and leading to the declaration of significant linkage peaks were no such peaks should be found. The simulation study also shows that the proposed bootstrap procedure seems to produce slightly inflated type I error rates as the signal-to-noise ratio increases. This problem is likely due to the fact that the markers used for mapping are also used to estimate the length of the segments shared identical by descent but also it can be associated with a stronger departure from exchangeability as the ratio increases. In any case, the problem deserves further scrutiny. The proposed bootstrap procedure for mixed models is quite general and can easily be adapted to non-genetic problems.

## Funding

This work has been supported by the Canadian Institutes of Health Research.

### Conflict of interest statement

The authors declare that the research was conducted in the absence of any commercial or financial relationships that could be construed as a potential conflict of interest.
